# Elimination of all redundant climbing fiber synapses requires granule cells in the postnatal cerebellum

**DOI:** 10.1038/s41598-018-28398-7

**Published:** 2018-07-03

**Authors:** Yannick Bailly, Sylvia Rabacchi, Rachel M. Sherrard, Jean-Luc Rodeau, Valérie Demais, Ann M. Lohof, Jean Mariani

**Affiliations:** 1 Intracellular Membrane Trafficking in the Nervous and Neuroendocrine System, INCI, CNRS UPR3212, Universite de Strasbourg, Strasbourg, France; 2 Sorbonne Université, CNRS UMR 8256, Biological Adaptation and Ageing, B2A, 75005 Paris, France; 3 APHP, DHU FAST, Institut de la longévité, 94205 Ivry-Sur-Seine, France; 4 Nociceptive Signalling in the Spinal Cord, CNRS UPR3212, Universite de Strasbourg, Strasbourg, France; 5 Plateforme d’Imagerie In vitro, CNRS UPS 3156 Universite de Strasbourg, Strasbourg, France; 6 Present Address: BiogenIdec, Inc., Cambridge, Massachusetts 02140 USA

## Abstract

Different afferent synapse populations interact to control the specificity of connections during neuronal circuit maturation. The elimination of all but one climbing-fiber onto each Purkinje cell during the development of the cerebellar cortex is a particularly well studied example of synaptic refinement. The suppression of granule cell precursors by X irradiation during postnatal days 4 to 7 prevents this synaptic refinement, indicating a critical role for granule cells. Several studies of cerebellar development have suggested that synapse elimination has a first phase which is granule cell-independent and a second phase which is granule cell-dependent. In this study, we show that sufficiently-strong irradiation restricted to postnatal days 5 or 6 completely abolishes climbing fiber synaptic refinement, leaving the olivo-cerebellar circuit in its immature configuration in the adult, with up to 5 climbing fibers innervating the Purkinje cell in some cases. This implies that the putative early phase of climbing fiber synapse elimination can be blocked by irradiation-induced granule cell loss if this loss is sufficiently large, and thus indicates that the entire process of climbing fiber synapse elimination requires the presence of an adequate number of granule cells. The specific critical period for this effect appears to be directly related to the timing of Purkinje cell and granule cell development in different cerebellar lobules, indicating a close, spatiotemporal synchrony between granule-cell development and olivo-cerebellar synaptic maturation.

## Introduction

The refinement of neuronal circuits during development is critical to their subsequent function; abnormalities in this process can result in neurodevelopmental disorders (eg autism spectrum disorder, schizophrenia, etc). In many brain regions, the correct number of contacts between pre- and postsynaptic partners depends upon the elimination of redundant synapses established at earlier developmental stages^1–7^. This afferent competition requires correct function and maturation of both synaptic partners^7–11^.

The rodent olivo-cerebellar path is a particularly informative and well-studied model of synaptic refinement^12,13^. In the cerebellar cortex, the interactions between parallel fiber and climbing fiber synapses on Purkinje cells demonstrate heterosynaptic competition during development^8^. The mature monoinnervation of Purkinje cells (PCs) by their climbing fibre afferents (CFs) from the inferior olivary nucleus^14,15^ is preceded by a transient stage of multiple innervation. In the rat, recordings in the latest-developing lobules of the posterior cerebellar vermis (lobules VI to VIII), showed CF synapse redundancy reaching a maximum of up to 5 CFs per PC (about 3.5 on average) on postnatal day 5 (P5) and then regressing until monoinnervation is established on P14-15^16–19^.

Cerebellar granule cells (GCs) are crucial for elimination of supernumerary CF synapses: if their parallel fiber (PF) synapses are absent or abnormal, CF synapse elimination is perturbed^12,20^. A previous study of CF innervation in which granule cell precursors (GCPs) were suppressed by postnatal X-irradiation (200 rads at P0, P3, P5, P7, P10, P12 and P14) seemed to indicate that GCs are not involved in the early part of CF synapse elimination. Recordings from animals undergoing this protocol sampled at different days starting at P3 showed that CF regression occurred normally until P8, and no further regression of the supernumerary CF synapses occurred^16^. Thus, only the final phase, after P8, would require GCs and would be driven by PF activity. This model has been reinforced by experiments with transgenic mice having faulty PF postsynaptic signaling. In these mice, lacking the type-1 metabotropic glutamate receptor^21^ or one of its downstream signaling molecules (Gαq^22^, PLCβ4^23^, PKCγ^24,25^), CF synapse elimination is not completely blocked, with 30–40% of PCs multiply-innervated and with an average of 2.5 CFs/PC in the adult. This is less than the average of 3.5–4 CFs per PC seen in early development; thus these observations of partially-disrupted CF synapse elimination supported the hypothesis of an early phase of GC-independent synapse elimination^20,25^.

Suppression of GCPs by X-irradiation allows the experimenter to control precisely the time and the extent of GCP suppression while leaving already-differentiated GCs not visibly affected^26,27^ (see Materials and Methods section). With this approach, Mariani *et al*.^28^ showed that the GCPs suppressed by irradiation during a critical period (P4-P7) are necessary to initiate CF synapse elimination; their protocol allowed maintenance of multi-innervation at almost the maximum developmental level (3.5 ± 0.85 CFs per PC^18^).

In this study, we (a) defined more precisely the critical period when GCs are necessary to produce total CF elimination; and (b) identified that this critical period depends upon the stage of PC and GC maturation, not simply chronology. We were able to identify degranulating irradiation protocols which allowed retention of up to 5 CFs per PC, which is the maximum level occurring during early postnatal development. We thus argue that the proposed early phase of synapse elimination cannot be independent of interactions with GCs or GCPs. If it were GC-independent, partial synapse elimination should have taken place even with complete GC loss. Our results indicate that the entire process of CF synapse elimination depends on the presence of a minimal GC population during a critical postnatal time window.

## Results

**Figure 1:**
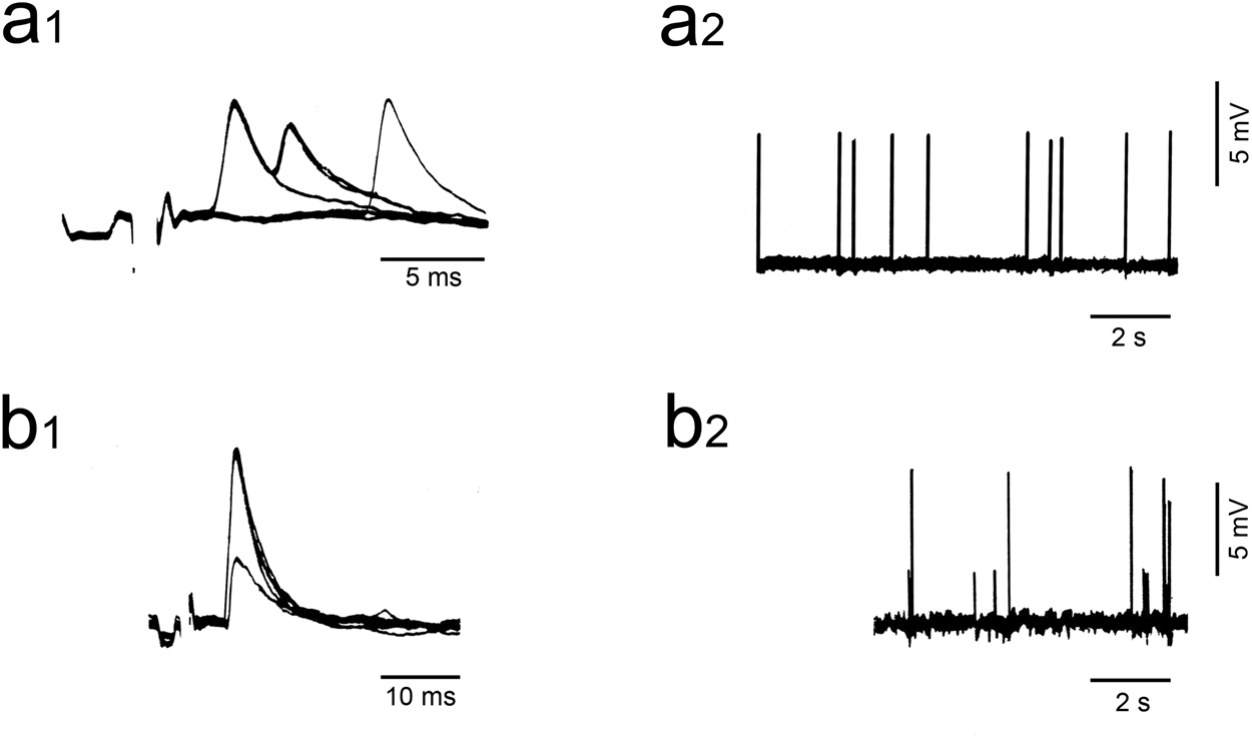
Intracellular recordings of Purkinje cell (PC) activity in X-irradiated rats. (**a1,2**) Typical all-or-none climbing fiber excitatory postsynaptic potential (CF-EPSP) in a PC innervated by a single CF either in response to stimulation (**a1**) or spontaneously (**a2**). A slow scanning speed (a2) reveals constant CF-EPSP amplitude. (**b1,2**) CF-EPSPs recorded from another PC and showing 2 CF-EPSP steps either when stimulation was gradually increased (**b1**) or during recording of spontaneous activity (**b2**). a1 and b1 show superimposed sweeps.

We measured multiple CF innervation in adult animals irradiated during the postnatal period using electrophysiological recording of PC activity (Fig. 1). For each experimental group, we determined (1) the percentage of PCs multiply innervated by CFs; and (2) the index of multiple innervation *m*, which is the number of steps (from 1 to 5) in spontaneous and evoked CF-EPSPs. The data we present support the hypothesis that the GCPs which are vulnerable to radiation damage around P5 are most important in the process of synapse elimination in the late-developing lobules VI-VIII.

**Figure 2:**
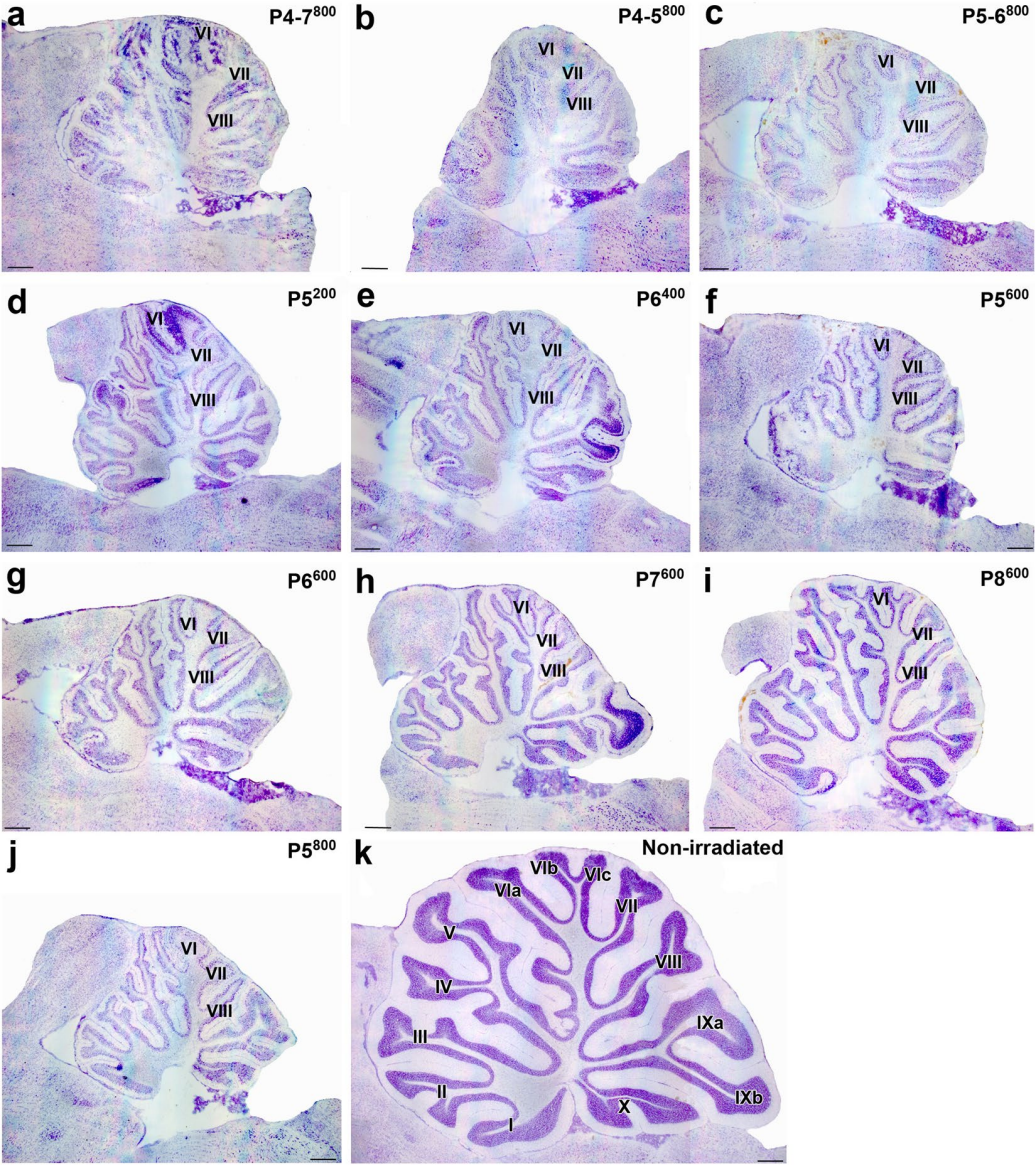
Representative thionin-stained parasagittal cryostat sections of adult cerebella from neonatally X-irradiated rats. (**a**–**j**) X-irradiated cerebella from Group P4-7^800^ (**a**), P4-5^800^ (**b**), P5-6^800^ (**c**), P5^200^ (**d**), P6^400^ (**e**), P5^600^ (**f**), P6^600^ (**g**), P7^600^ (**h**), P8^600^ (**i**), P5^800^ (**j**). The late-developing lobules VI, VII and VIII display the most extreme atrophy. (**k**) Non-irradiated control cerebellum. Scale bars = 500 µm.

**Figure 3:**
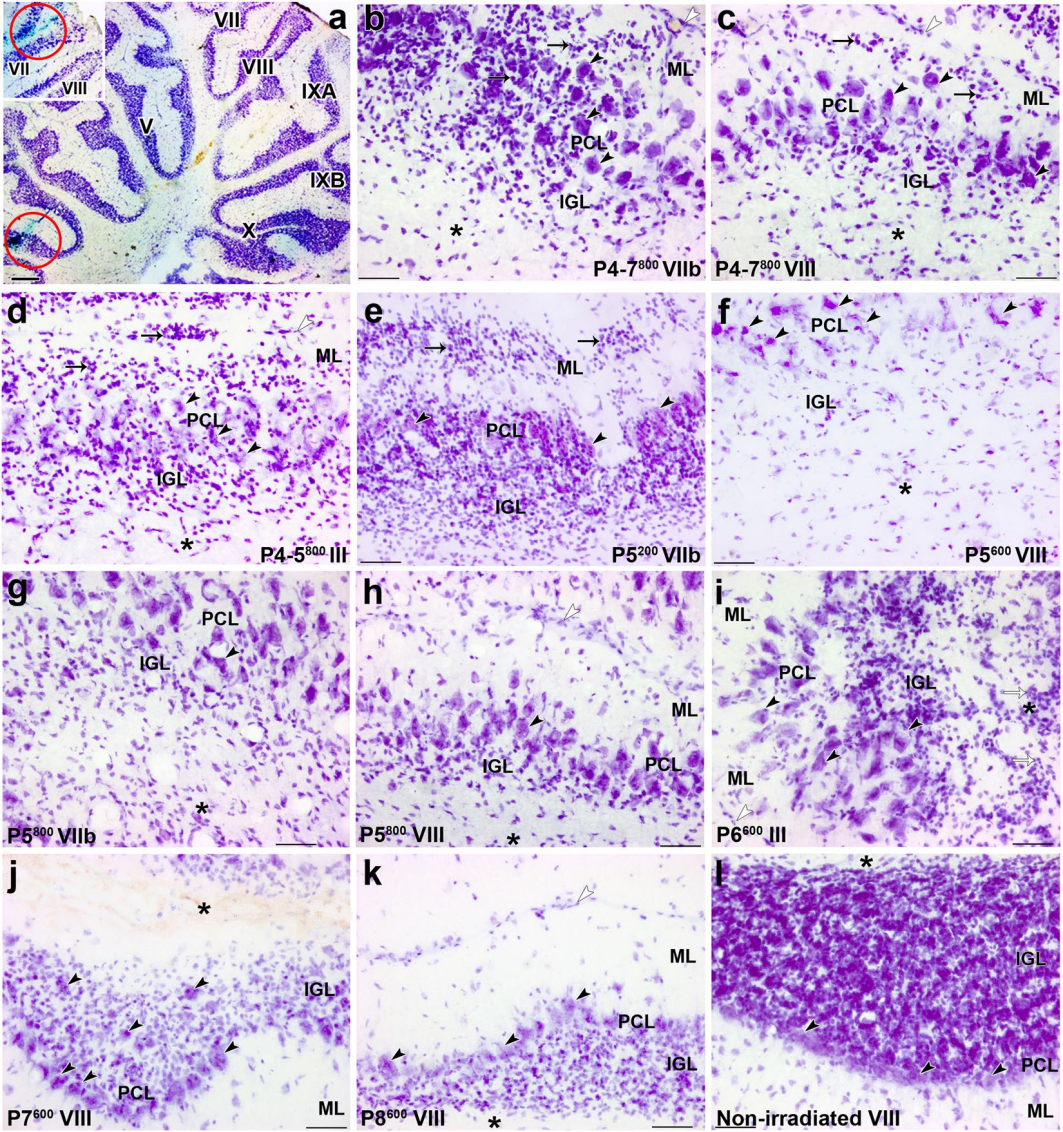
Abnormal histological features in the cerebellar cortex of adult rats, induced by postnatal X-irradiation. (**a**) The tracks of the recording microelectrode can be reconstructed from the position of the two deposits of Fast green (red circles visible in lobules III (a) and VII (inset). (**b**–**i**) Abnormal arrangement of PCs (black arrowheads) in multiple layers (PCL) in lobules VIIb (**b**) and VIII (**c**) in Groups P4-7^800^, lobules III (**d**) in Group P4-5^800^, lobules VIIb (**g**) and VIII (**h**) in Group P5^800^ and lobule III (**i**) in Group P6^600^. PCs are more or less normally distributed in a monolayer in lobule VIII in Groups P7^600^ (**j**), P8^600^ (**k**) as well as in the non-irradiated control cerebellum (**l**). Ectopic granule cells (arrows) are located in the molecular layer (ML) and PCL in lobules VIIb (**b**) and VIII (**c**) in Groups P4-7^800^ and P5^200^ (**e**) and in lobule III (**d**) in Group P4-5^800^. In the atrophied internal granular layer (IGL), granule cells are extremely depleted in Groups P4-7^800^ (**b**,**c**), P4-5^800^ (**d**), P5^800^ (**g**,**h**), P5^600^ (**f**) and P6^600^ (**i**). These cells are partly restored in the IGL in Groups P7^600^ (**j**) and P8^600^ (k). Abnormal location of granule cells (white arrows) in the white matter (*) is seen in the lobule III in Group P6^600^ (**i**). White arrowheads indicate the surface of the cerebellar cortex in (**b**,**c**,**d**,**h**,**i**,**k**). Scale bars = 500 µm in a, 50 µm in b-l.

**Figure 4:**
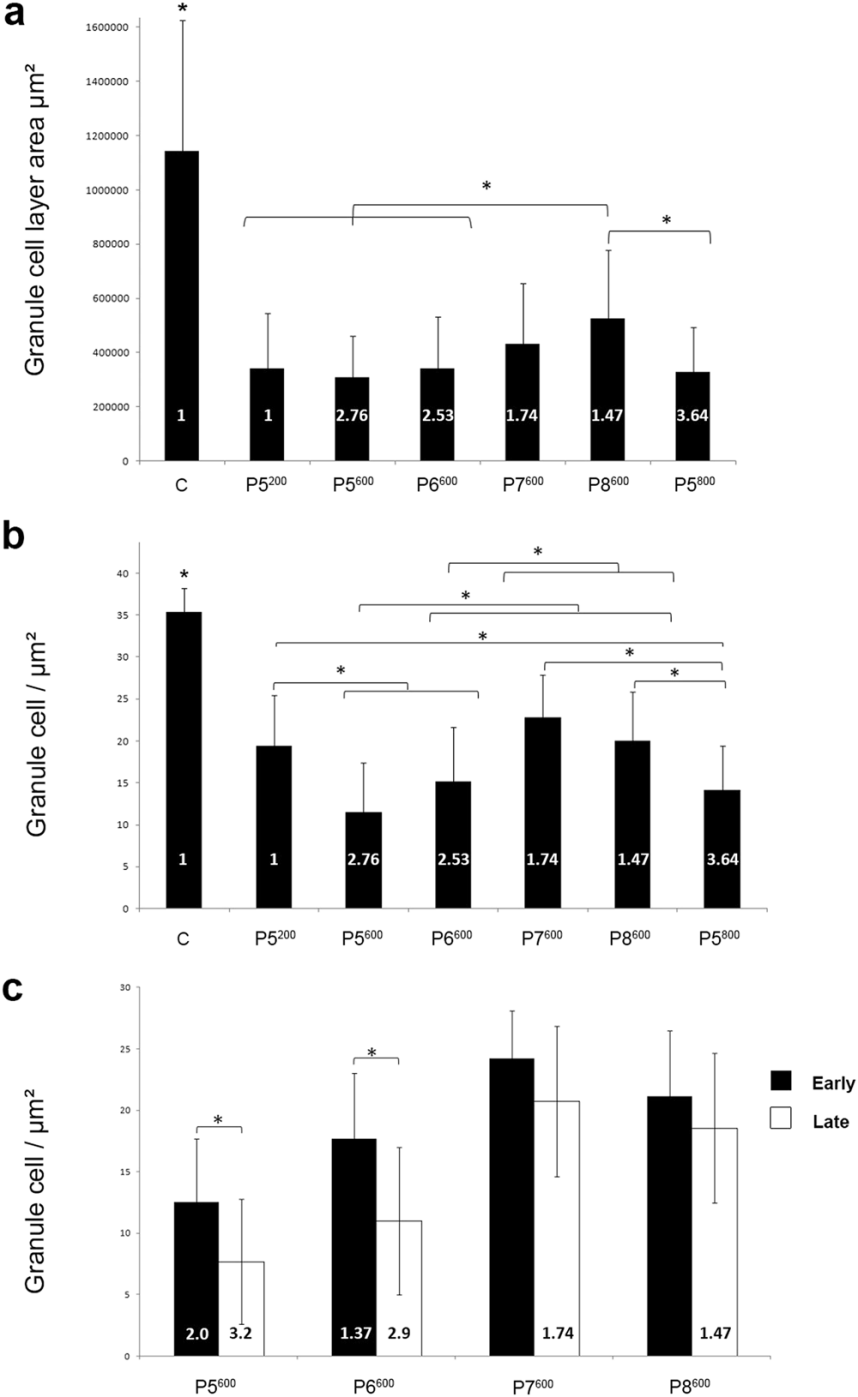
Quantitative analysis of granule cell loss in the cerebellum of postnatally X-irradiated adult rats. (**a**) Granule cell area is significantly reduced in the cerebellum of X-irradiated rats compared to the cerebellum of non-irradiated control rats (C). In the P8^600^ irradiated cerebella, granule cell loss is significantly less than in the cerebella irradiated at the critical days P5-P6 in Groups P5^200^, P5^600^, P5^800^ and P6^600^. Bars show mean ± S.D. in all graphs. (**b**) Granule cell density is significantly decreased in the cerebellum of all X-irradiated groups compared to the cerebellum of non-irradiated control rats (C). A significant further decrease in granule cell density is also obtained with doses above 200 rads at P5 (P5^600^, P5^800^) and P6 (P6^600^). A dose of 600 rads produces significantly greater granule cell loss when delivered at P5 (P5^600^) or P6 (P6^600^) than at P7 (P7^600^) or P8 (P8^600^). (**c**) In the cerebellum of rats irradiated with 600 rads, granule cell loss is significantly greater in the late-developing lobules VI-VIII than in the early-developing lobules IX-X after irradiation at P5 (P5^600^) or P6 (P6^600^) but not at P7 (P7^600^) or P8 (P8^600^). The values of mean index of CF innervation *m* are indicated within the bars. *P ≤ 0.01.

**Figure 5:**
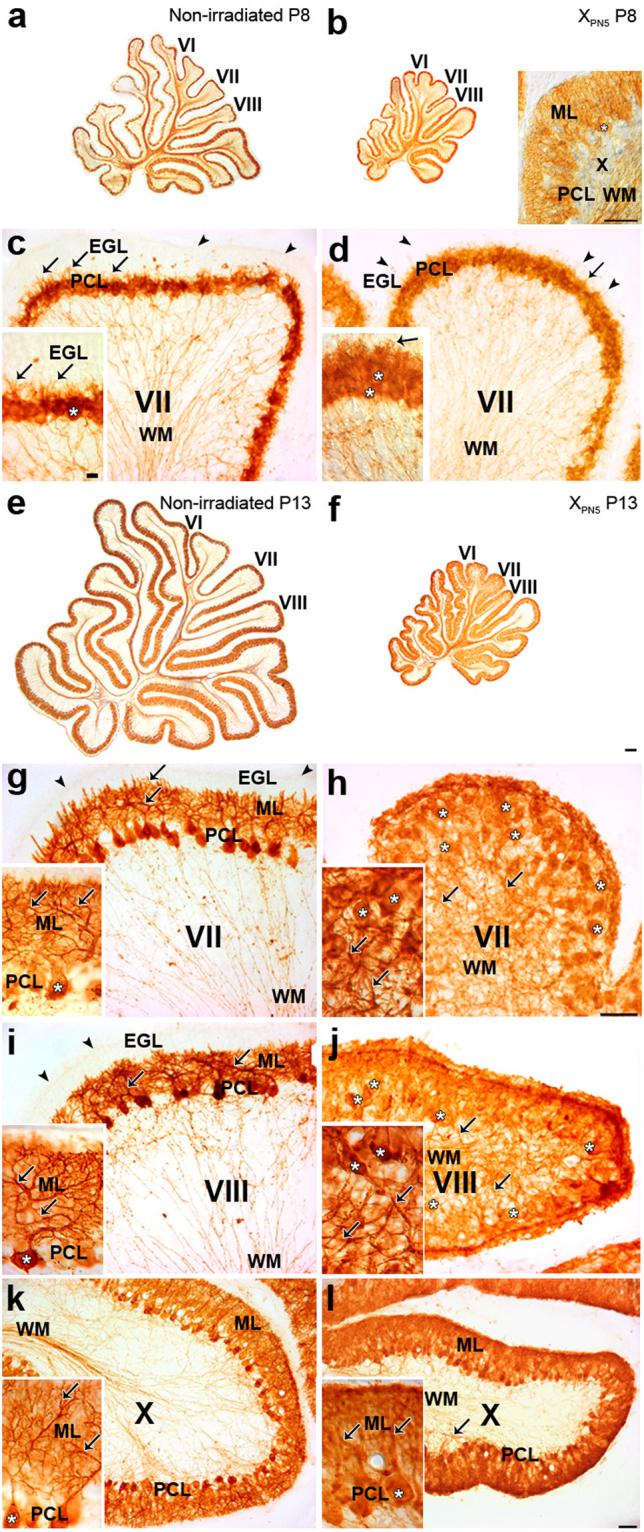
CaBP–immunohistochemical staining of Purkinje cells in the developing cerebellum of normal and postnatally X-irradiated rats from Group P5^600^. (**a**,**b**,**e**,**f**) Low magnification of sagittal sections of the cerebellar vermis of control (**a**,**e**) and X-irradiated Group P5^600^ (**b**,**f**) rats at P8 (**a**,**b**) and P13 (**e**,**f**). Insets b, c, d, g-l, High magnifications of sagittal sections of the late-developing lobules VII (**c**,**d**,**g**,**h**) and VIII (**i**,**j**) to compare with early-developing lobule X (**b**,**k**,**l**) in the cerebellar vermis of normal rats at P8 (**c**) and P13 (**g**,**i**,**k**) and Group P5^600^ X-irradiated rats at P8 (**b**,**d**) and P13 (**h**,**j**,**l**). PCs (white asterisks) are already disorganized in the 8-day-old X-irradiated lobule VII in (**d**). Short PC dendrites (arrows) are visible in the nascent molecular layer in the normal and X-irradiated P8 lobule VII. PC dendrites have abnormally grown in the axial white matter (WM) of the X-irradiated late-developing lobules VII (**h**) and VIII (**j**) and, to a much lesser extent in early-developing lobule X (**l**) at P13. Immunostained PC axons are the only visible structures in WM of the cerebellar lobules of normal (**c**) and X-irradiated (**d**) P8 rats as well as of the normal P13 (**g**,**i**,**h**) rats. In the low power image (**b**) and in each medium-power image in (**c**,**d**,**g**–**l**), the inset shows higher magnification of PCs. White asterisks indicate the somata of representative PCs. Arrowheads show the surface of the external granular layer (EGL) in (**c**,**d**,**g**,**i**). The EGL has virtually disappeared in the X-irradiated late-developing lobules VII (**i**) and VIII (**j**) at P13. ML, molecular layer; PCL, Purkinje cell layer. Scale bars in insets b, c, d, g–l = 50 µm. Scale bar in f = 200 µm for (**a**,**b**,**e**,**f**). Scale bar in inset c = 10 µm for insets (**c**,**d** and **g**–**l**).

Irradiation protocols targeted the first postnatal week because at this stage the ventral-to-dorsal gradient of development in the cerebellum allows us to observe effects of irradiation on relatively mature (early-developing) ventral lobules and less mature (late-developing) dorsal lobules in the same animal. At these stages, many GCs will already have been generated in the early-developing ventral regions^27^ and thus degranulation seen in the adult will be less. Morphology observed after the electrophysiological experiments showed more GC loss and more severe and abnormal PC positioning (multilayering) in late-developing lobules VI, VII and VIII than in earlier-developing lobules I-V and IX-X (Figs 2, 3, 4 and 5).

To examine the relationship between GC loss and the multiple innervation induced by postnatal X-irradiation, GC density and the area occupied by the granule cell layer (GCL) were measured for some groups exposed to a single irradiation dose (Fig. 4). The GCL was significantly decreased in all groups compared to controls. Within these reduced GCL areas, GC densities varied between groups, and underscored the importance of irradiation age and dose in determining the relationship between GC loss and persistent CF multiple innervation.

For all experimental groups we concentrated our electrophysiological measurements of multi-innervation on the late-developing lobules, but in some experimental groups we were also able to compare it with multi-innervation in earlier-developing lobules. For simplicity of analysis we have grouped the PCs in the late-developing lobules together and the PCs in the early-developing lobules together.

We aimed (1) to define the critical period during which GCP suppression is most disruptive to CF synapse elimination and (2) to understand the interaction between the degree of degranulation and PC development in this synaptic refinement.

### What is the critical period for granule cell loss to maintain multiple innervation?

We first confirmed the morphological and electrophysiological observations from a previous study^28^ which demonstrated that a large irradiation dose applied over 4 days led to highly-disrupted CF synapse elimination and much reduced cerebellar size (Fig. 2a) when compared to non-irradiated adult cerebellum (Fig. 2k). In Group P4-7^800^ (that is, irradiated on days P4-7 for a total dose of 800 r; Table 1), granule cell depletion occurred in all lobules and an immature multilayered arrangement of Purkinje cells was particularly pronounced in late-developing lobule VII (Fig. 3b). An ectopic layer of arrested granule cells was also present in the deep part of the molecular layer throughout the anterior cerebellum and in lobules VIII and IX (Fig. 3c).

**Table 1:** Different schedules of postnatal (P) X-irradiation used.

Groups	PDays					D (r)	n
	4	5	6	7	8		
P4-7^800^	200	200	200	200		800	**11**
P4-5^800^	400	400				800	**7**
P5-6^800^		400	400			800	**8**
P5^200^		200				200	**2**
P5^600^		600				600	**10**
P5^800^		800				800	**5**
P6^400^			400			400	**1**
P6^600^			600			600	**12**
P7^600^				600		600	**3**
P8^600^					600	600	**5**

D is the total dose in rads (r) received (100 r = 1 Gy) and n is the number of rats in each experimental group.

**Figure 6:**
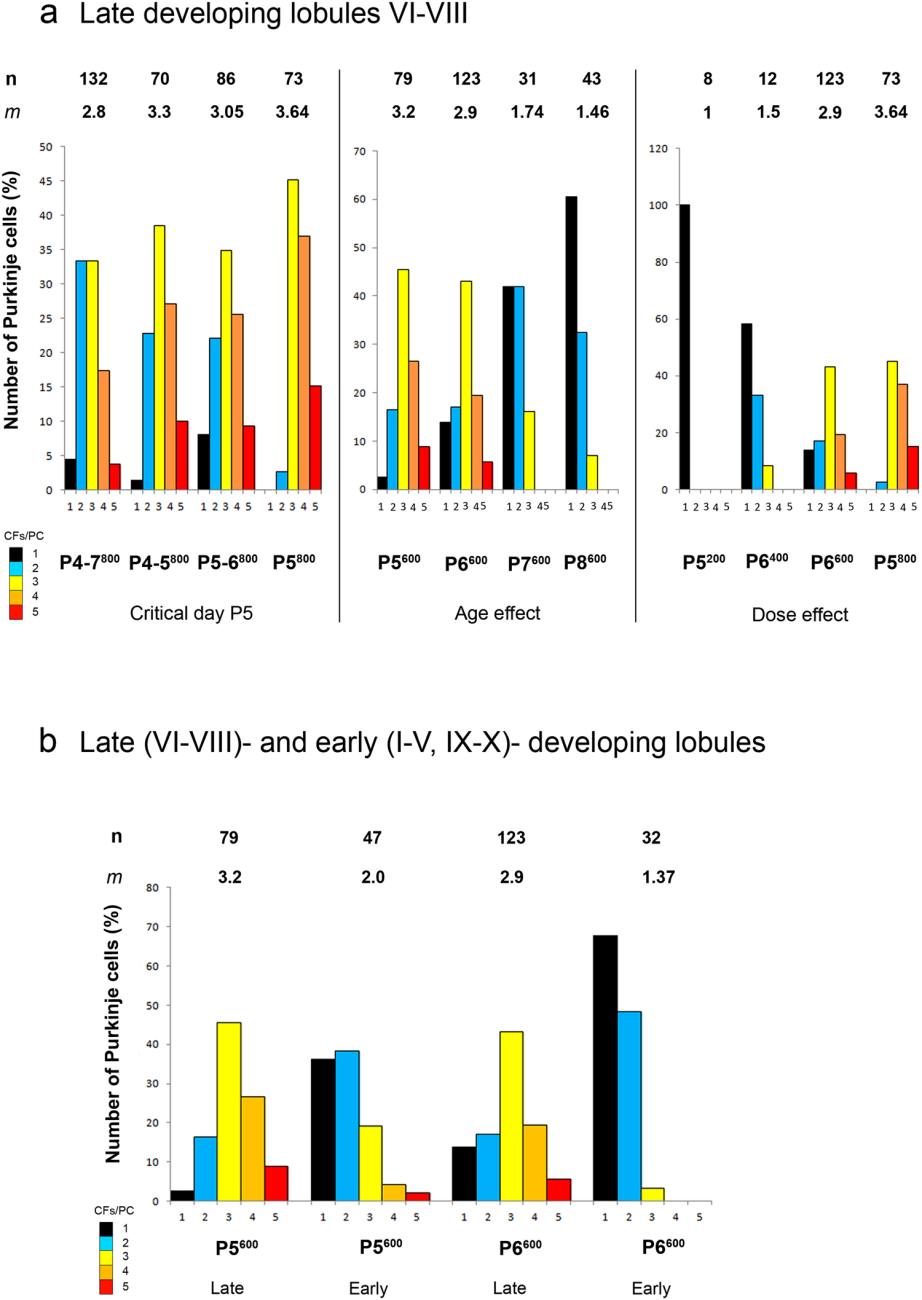
The number of cells studied (n) and the mean index (*m*) of multiple CF innervation in the corresponding groups are indicated in upper lines. The number of PCs mono-innervated and multi-innervated by 2 to 5 CFs is given in percent of the total number of recorded PCs and color-coded from black (1 CF/PC) to red (5 CFs/PC). (**a**) Distribution of CFs per PC in the late-developing cerebellar lobules VI, VII and VIII for different X-irradiated groups. Left. A single 800 r X-irradiation at the critical postnatal day 5 (P5^800^) maintains a greater index of multiple innervation than the same dose delivered over 2 days (2 × 400 r; P4-P5^800^ and P5-P6^800^) or 4 days (4 × 200 r; P4-P7^800^). Middle. The irradiation effect depends on development stage. Multiple innervation decreases when a single 600 r dose is given at progressively later days (Groups P5^600^, P6^600^, P7^600^ and P8^600^). Right. Decreasing the X-ray dose delivered over the P5-6 period decreases multiple innervation retained in the adult cerebellum. In Group P5^200^, the elimination process was completed normally; increasing doses increased retained CF multiple innervation. (**b**) Distribution of CFs per PC in the late- (VI-VIII) and early- (I-V, IX, X) developing cerebellar lobules of X-irradiated rats of Groups P5^600^ and P6^600^. A single 600 r X-irradiation at P5 (Group P5^600^) blocks CF synapse elimination more effectively than the same dose at P6 (Group P6^600^) in the late-developing and in the early-developing lobules. In both cases, this dose more efficiently blocks synapse elimination in the late-developing lobules than in the early-developing lobules.

Multiple innervation was high in animals receiving this dose. Of the 133 PCs recorded from 11 rats, 126 (94.7% overall, including 99% of PCs in lobule VII) were innervated by multiple CFs. The mean index of innervation (*m*) was 2.81 ± 0.08 (Table 2). The number of CFs for a substantial proportion of these PCs was 4 or 5 (Fig. 6a-left). These electrophysiological data are in agreement with those obtained previously using the same X-irradiation schedule (*m* = 2.73 ± 0.07 all lobules^28^).

**Table 2:** Percentage of multiply innervated Purkinje cells (%) and mean index (*m*) of Purkinje cell innervation by climbing fibers (mean ± S.E.M.) in the total sample of PCs and cerebellar lobules from 10 groups of postnatally X-irradiated adult rats.

*Group*	P4-7^800^	P4-5^800^	P5-6^800^	P5^200^	P5^600^	P5^800^	P6^400^	P6^600^	P7^600^	P8^600^
*Number of rats*	11	7	8	2	10	5	1	12	3	5
*Total sample of PCs*										
Nb PC	133	72	86	16	126	73	14	159	31	43
%	94.7	97.2	91.9	0.0	84.9	100	35.7	76.1	58.1	39.5
*m*	2.81 ± 0.08	3.17 ± 0.12	3.06 ± 0.12	1.00	2.76 ± 0.10	3.64 ± 0.09	1.43 ± 0.17	2.53 ± 0.09	1.74 ± 0.13	1.47 ± 0.10
*Lobules I-V*										
Nb PC	0	0	0	5	0	0	2	0	0	0
%	/	/	/	0.0	/	/	0.0	/	/	/
*m*	/	/	/	1.00	/	/	1.00	/	/	/
*Lobule VI*										
Nb PC	8	2	3	0	1	18	4	3	13	3
%	62.5	100	100	/	100	100	25.0	100	30.8	0.0
*m*	2.00 ± 0.33	3.00 ± 1.00	2.33 ± 0.33	/	4.00	3.44 ± 0.15	1.25 ± 0.25	2.67 ± 0.33	1.31 ± 0.13	1.00
*Lobule VII*										
Nb PC	84	55	71	6	60	47	8	85	18	30
%	98.8	100	93.0	0.0	98.3	100	50.0	95.3	77.8	46.7
*m*	2.95 ± 0.10	3.36 ± 0.13	3.24 ± 0.13	1.00	3.22 ± 0.11	3.74 ± 0.12	1.63 ± 0.26	3.20 ± 0.10	2.06 ± 0.17	1.57 ± 0.12
*Lobule VIII*										
Nb PC	40	13	12	2	18	8	0	34	0	10
%	95.0	92.3	83.3	0.0	94.4	100	/	61.8	/	30.0
*m*	2.73 ± 0.13	2.62 ± 0.24	2.17 ± 0.21	1.00	3.22 ± 0.26	3.50 ± 0.33	/	2.03 ± 0.16	/	1.30 ± 0.15
*Lobule IX*										
Nb PC	1	2	0	0	34	0	0	32	0	0
%	0.0	50.0	/	/	61.8	/	/	43.8	/	/
*m*	1.00	1.50 ± 0.50	/	/	2.06 ± 0.18	/	/	1.47 ± 0.10	/	/
*Lobule X*										
Nb PC	0	0	0	3	13	0	0	5	0	0
%	/	/	/	0.0	69.2	/	/	40.0	/	/
*m*	/	/	/	1.00	1.77 ± 0.17	/	/	1.40 ± 0.24	/	/

Next we narrowed the period of irradiation to 2 days within this previously-defined critical period. Groups P4-5^800^ and P5-6^800^ received the same total dose of irradiation as in Group P4-7^800^. At the adult stage in these animals, we observed severe cerebellar atrophy (Fig. 2b,c), extreme depletion of the granule cell population with PCs in several layers throughout the cerebellum, and ectopic granule cells in all lobules (Fig. 3d). These effects were more severe in Group P4-5^800^ (Fig. 2b) than in Group P5-6^800^ (Fig. 2c). Table 2 indicates that in the late-developing lobules, where multi-innervation was highest, PCs in Group P4-5^800^ (*m* = 3.21 ± 0.11) and in Group P5-6^800^ (*m* = 3.05 ± 0.12) had similar levels of multi-innervation (Kruskal-Wallis p > 0.05). Again, it was possible to find PCs innervated by the developmental maximum of 5 CFs (Fig. 6a)^16,18,19^.

Finally, since P5 was common to both these previous groups, we applied the high dose of irradiation on this single day to maximize the degranulation effect. These cerebella (Group P5^800^) were severely atrophic (Fig. 2j), with considerable granule cell loss and PC multilayering throughout the cerebellum (Fig. 3g,h). All recorded PCs from this group were from the dorsal late-developing lobules and were innervated by multiple CFs, with *m* = 3.64 ± 0.09 (Table 2, Fig. 6a). Fifteen percent of these PCs were innervated by 5 CFs. This level of multi-innervation was significantly greater than that found for Groups P4-7^800^, P4-5^800^, or P5-6^800^ (Kruskal-Wallis and Dunn’s multiple comparisons, P < 0.05).

When GCL area (Fig. 4a) and GC density (Fig. 4b,c) were measured for any of the one-day doses of irradiation at P5 (200, 600, or 800 rads), GCL area was significantly decreased by all doses. GC density was decreased by the higher radiation doses compared to P5^200^, along with the increases in multiple innervation (Fig. 4b,c). This observation demonstrates that a low level of degranulation is not sufficient for retention of multiple CF innervation.

Taken together these data confirm the importance of the population of GCPs vulnerable to irradiation around P5 in triggering CF synapse elimination in the late-developing dorsal lobules. Although we can only speculate about the reason these particular GCPs and the GCs they will generate are critical for CF synapse elimination, P5 is the peak of multiple CF innervation, and thus the process of CF elimination begins at this stage.

### Interactions between granule cell loss and developmental timing in CF synapse elimination

In the experiments described above, a few PCs were recorded from earlier-developing ventral lobules (Table 2), which showed little CF multi-innervation despite appearing severely degranulated compared to control. We decided to explore in more detail the role of developmental stage in determining susceptibility of CF multiple innervation to GC loss, applying a single submaximal 600 r dose of irradiation on different days between P5 and P8, and examining electrophysiological and morphological aspects in late-developing (VI-VIII) and early-developing (IX-X) lobules. We measured GC density and GCL area, and carried out more detailed morphological analysis for some groups to understand the role of PC maturation in determining multiple innervation.

As expected from our results concerning the importance of the critical period around P5, granule cell loss, cerebellar atrophy, and PC multi-layering were pronounced throughout the cerebellar cortex after irradiation on P5 (Group P5^600^, Figs 2f and 3f), and reduced when irradiation took place on later days. The severity of these abnormalities decreased slightly for Group P6^600^ (Fig. 2g) and markedly for Groups P7^600^ (Figs 2h and 3j) and P8^600^ (Figs 2i and 3k). In some cases, we saw ectopic granule cells throughout white matter and cortex in lobules I-III (Fig. 3i).

Immunohistochemical staining for Calcium-binding D-28k protein (CaBP) of Group P5^600^ PCs on P8–i.e. three days after irradiation–revealed already the atrophy of the entire cerebellum (Fig. 5b) compared with the cerebellum of normal rats (Fig. 5a). This size difference was further increased by P13 due to the substantial development of the normal (Fig. 5e) but not of the X-irradiated (Fig. 5f) cerebellum. Similar morphological changes were observed in Group P6^600^ cerebella at the same ages (not shown). In both Groups P5^600^ and P6^600^, PC growth retardation was less pronounced in early-developing lobules I-V and IX-X than in late-developing lobules VI-VIII (Fig. 5).

A detailed analysis of irradiated cerebella from Group P5^600^ clearly demonstrated that X-irradiation impaired PC morphological development, and there were differences between late-developing and early-developing lobules. In the normal P8 cerebellum, PCs of the late-developing lobules VII (Fig. 5c) and VIII formed a monolayer and had a stellate-like shape with short apical processes, while in the early-developing lobule X, PCs were more mature, having already developed a prominent, apical dendritic tree with ramifications in the molecular layer. By contrast, in irradiated cerebella examined at P8, the PCs of lobules VII (Fig. 5d) and VIII appeared less mature; they were not distributed in a monolayer, and had short processes in all directions; in lobule X, PCs were similar to those found in controls (Fig. 5b inset).

These deficits in the maturation of PC morphology of P5^600^ animals were more marked at P13. In control P13 cerebella, all PCs were arranged in a monolayer and sent well-developed dendritic trees into the molecular layer, although PC dendrites were more developed in lobule X (Fig. 5k) than in lobules VII (Fig. 5g) and VIII (Fig. 5i), as expected. In contrast, as previously described in the cerebellar cortex of postnatally X-irradiated rats^27,29^, the PC layer of lobules VII (Fig. 5h) and VIII (Fig. 5j) in the irradiated cerebellum was markedly disorganized and PCs sent poorly-ramified dendrites towards the white matter core of the lobule. However, PCs in the early-developing lobules were much more mature. In lobule X for example, the PCs were still arranged in several layers yet sent correctly-oriented but atrophic dendrites from their apical pole in the normal upward direction (Fig. 5l). This likely reflects that the critical interaction between GCs and PCs has already taken place in this region, before the loss of GCPs induced by X-irradiation at P5.

Electrophysiological analysis of Group P5^600^ (Table 2, Fig. 6) showed pronounced multiple CF innervation in the late-developing lobules (97%, *m* = 3.23). Closer examination of the dorsal part of lobule VIII indicated that this severely-affected region had 100% multiple innervation and the highest mean index of 3.62 ± 0.038. In contrast, early-developing lobules were much less affected, with 64% of PCs multi-innervated and *m* = 1.98 ± 0.14 (*m* different for early-developing versus late-developing lobules, p < 0.0001, Kruskal-Wallis and Dunn’s multiple comparison) and only one maximally-innervated PC (Table 2, Fig. 6b).

Similar results were found when irradiation was applied on P6 (Group P6^600^; *m* not different for Groups P5^600^ and P6^600^ either in early-developing lobules, or in the late-developing lobules; Kruskal-Wallis and Dunn’s multiple comparisons, p > 0.05; Table 2, Fig. 6). In the early-developing lobule X, only 40% of the recorded PCs were innervated by multiple CFs with *m* = 1.40 ± 0.24 (Table 2, Fig. 6b). Significant differences in GC density were found between the early and late-developing lobules in these groups (P5^600^ and P6^600^) suggesting that the reduced multiple innervation in the early-developing lobules is the result of a greater density of already-generated GCs in these regions (Fig. 4c).

Treatment with the same dose of X-rays on subsequent days (Groups P7^600^ and P8^600^) confirms the interaction between maturation and susceptibility to maintained multi-innervation. Cerebella from Group P7^600^; Figs 2h and 3j) and Group P8^600^ (Figs 2i and 3k) showed clear atrophy and GC loss, although less than Groups P5^600^ (Figs 2f and 3f) and P6^600^, with most PCs in a monolayer in the late-developing lobules VI-VIII (Fig. 3j,k). Multiple CF innervation was much reduced compared to groups irradiated at earlier days with the same dose. In the late-developing lobules, only 58.1% of PCs were multi-innervated in Group P7^600^ (*m* = 1.74) and 39.5% in Group P8^600^ (*m* = 1.47) (Table 2, Fig. 6a-middle; *m* for late developing lobules in Groups P7^600^ and P8^600^ different from those in Groups P5^600^ and P6^600^; Mann Whitney, p < 0.0001). No PCs were innervated by more than 3 CFs in these cerebella. These differences in CF multiple innervation are paralleled by differing levels of degranulation. Although GCL area was similarly reduced in Groups P5^600^, P6^600^, P7^600^ and P8^600^ (Fig. 4a), measures of GC density showed that irradiation on the later days produced less degranulation (Fig. 4b). This is likely because fewer GCs remained to be produced by the GCPs still dividing at these later days. Finally, quantification of GC density in the P7^600^ and P8^600^ groups showed no differences between early- and late-developing lobules (Fig. 4c). This suggests that by P7/P8 enough GCs have been generated throughout the cerebellum to provide nearly-normal CF synapse elimination.

### Relation between dose of irradiation and index of multiple innervation

Comparison of different groups receiving different amounts of irradiation during a similar time period (P5-P6) clearly demonstrates the dose-dependence of the retained multiple CF innervation. In rats receiving X-irradiation at P5, the key stage for GC-dependent induction of CF synapse elimination, a single 200 r X-irradiation did not produce retained multiple CF innervation in the adult (Group P5^200^; late-developing lobules VII-VIII, 0% of 16 PCs, *m* = 1, Table 2, Fig. 6a-right). Interestingly, the GCL area and GC density were considerably reduced in this group (Figs 2d, 3e and 4a,b) compared to control (Figs 2k, 3l and 4), but this level of degranulation appears to be “sub-threshold”, in that enough GCPs survive in the external granular layer and subsequently generate enough GCs to trigger CF synapse elimination. Thus even during the critical period around P5, the suppression of GCPs will not interfere with complete CF synapse elimination if the irradiation dose is low.

Nevertheless, level of degranulation similar to that in Group P5^200^ was induced in the P7^600^ and P8^600^ Groups with a significant effect on synapse elimination (P7^600^: 58.1% of 31 PCs, *m* = 1.74 ± 0.13; P8^600^: 39.5%, *m* = 1.47 ± 0.10) underscoring that reduced GCL area and GC density in the postnatally irradiated adult cerebellum are not linearly related to reduced CF synapse elimination.

Increasing the irradiation dose at the same day confirms the dose-dependency of CF synapse elimination (Fig. 6a-right). For example, a single 400 r X-irradiation on P6 (Group P6^400^; 35.7% of 14 PCs, *m* = 1.43 ± 0.17) was much less effective than 600 r on the same day (Group P6^600^; 76.1% of 159 PCs, *m* = 2.53 ± 0.09), which almost completely prevented CF synapse elimination (Table 2; Wilcoxon test, p = 0.007). This difference was even clearer in the late-developing lobules, with *m* = 2.9 after 600 r compared to *m* = 1.5 after 400r (Fig. 6a-right). In addition, a single 800 r dose delivered on P5 maintained a higher degree of multiple innervation than a single dose of 600 r on the same day (in lobules VII and VIII, Group P5^800^ versus Group P5^600^: Kruskal-Wallis, p = 0.0085, Table 2).

All these results support the critical interaction of developmental stage and suppression of GCPs in triggering CF synapse elimination. Suppressing the GCPs when a PC has reached a certain stage of maturation will have little or no effect on CF multi-innervation, despite reducing the GC population found in the adult. This can be seen either in the same animal in early- and late- developed lobules (Fig. 6b) or by looking at the same lobules irradiated at different developmental dates (Fig. 6a-middle).

## Discussion

Previous studies of synaptic refinement in the olivocerebellar pathway showed that the end of the first post-natal week is critical for the granule-cell-dependent effects on CF synapse elimination in the rat^28–30^. In this study, we have better defined this critical period, and we have shown that the *entire* process of CF synapse elimination requires the presence of a sufficient number of granule cells interacting with PCs at a specific stage of maturation. This contrasts with previous suggestions that the early phase of synapse elimination is granule cell-independent.

Although X-irradiation can produce apoptosis in glial cells as well as in neurons^27,31^, the X-ray dose-dependency between degranulation and retained CF-PC multiple innervation suggests that the main effect of irradiation is due to this suppression of GCPs^16,28,30,32,33^. This observation is also consistent with other animal models which suffer from early loss of GCPs and present abnormal retention of multiple CF innervation into adulthood: reeler^34^ or weaver^35^ mutant mice, or neonatal viral infection in ferrets^36^.

### Relation between time of irradiation, PC maturation, and multiple innervation

A previous study showed that 800 r delivered over days 4–7 gave a multi-innervation index (*m*) of 2.75–3 climbing fibers per Purkinje cell (CFs/PC)^28^ a result we confirmed here (Group P4-7^800^). This period can be even more restricted, since we obtained a mean index of multiple innervation (*m*) statistically equivalent to that maintained in Group P4-7^800^ when the same total dose was delivered on either P4-5 or P5-6, and *m* was slightly greater when the entire 800 r dose was administered on P5. Therefore the critical period can be reduced to one or two days if an equivalent total dose of 800 r is given. Postnatal day 5 is the day for which multiple CF innervation is maximal in the rat^16–19^, and thus elimination begins at this point, which may be determine the importance of this specific day.

It is important to note that irradiation protocols which lead to considerably fewer granule cells in the adult animal may not result in the same index of retained multiple innervation, depending on the age at which degranulation is induced. Cerebellar histology for Groups P5^600^, P6^600^, P7^600^ and P8^600^ shows substantial atrophy of the internal granular layer (IGL) in dorsal lobules in all four groups (Fig. 3f,i–k) compared to non-irradiated control (Fig. 3l), although irradiation at P5 or P6 produced greater atrophy (Fig. 4). However, multiple innervation is reduced in cerebella irradiated at P7-8 (Groups P7^600^ and P8^600^) compared to cerebella irradiated at P5-6 (Groups P5^600^ and P6^600^), both in percentage of PCs multiply innervated and the index *m* (Fig. 6a-middle). The likely explanation is that the GCPs suppressed by irradiation around P5 in the late-developing lobules are critical for triggering synapse elimination. It seems that the already-differentiated GCs present at P5 are not necessary for the CF synapse elimination process, since this process is completely blocked despite their presence. While irradiation of slightly older animals (P7-8) produces severe atrophy of the IGL in the adult by suppressing the later-generated GCs (Fig. 4b), this GCP suppression is too late to cause retention of maximal CF multiple innervation.

### Regional differences in developmental timing, and consequences for multiple innervation

The chronology of GC generation and maturation differs in the different cerebellar lobules^27^. The maturation state of Purkinje cells and the cerebellar circuit in these different lobules will determine how CF synapse elimination is altered by GC loss.

In our study, early developing lobules (I-V, IX-X) showed less granule cell loss, less atrophy and PC multilayering, and less growth retardation compared to the late-developing lobules (VI-VIII). Since multiple CF innervation was also much higher in these late-developing lobules, these observations confirm that granule cell loss, PC growth impairment and synapse elimination blockade are closely correlated. In the earlier-developing lobules, the “critical” GCs seem to have already had their effect on CF synapse elimination before the time of irradiation.

### A granule-cell-independent phase of synapse elimination?

Previous studies^16,37^ have suggested that CF synapse elimination proceeds in two distinct phases: an early phase which is independent of PF-PC synapse formation; and a late phase that depends critically on PF-PC synapses. Because PC innervation by multiple CFs appeared to regress normally between P5 and P8 in the cerebella of rats receiving repeated doses of 200 r (on P0, 3, 5, 7, 10, 12 and 14), Crepel *et al*.^16^ suggested that this early phase of CF synapse elimination was GC-independent. Although these repeated irradiations ultimately eliminated the majority of GCs, the protocol spares enough early GCPs, which continue to generate GCs during the critical period around P5. Consistent with this view, Altman *et al*.^38^ showed that following early irradiation (200 r on P0 and P1), many GCs are present both at P8 and in the adult. More recently, nestin-expressing glial progenitors issued from the PC layer have been demonstrated to switch their fate to regenerate GCPs following a single 400 r X-irradiation at P1^39^. Whatever their origin, these GCPs could generate enough GCs to trigger partial CF synapse elimination: the multiple innervation index of animals irradiated with 200r daily during the 3 first postnatal days was *m* = 1.27 ± 0.04^28^.

In contrast, in our study a strong degranulating X-irradiation protocol (i.e. P5^800^) fully blocked the putative early and late phases of CF regression. In late-developing lobules VI-VIII, more than 3.6 CFs per PC remained after a single 800 r irradiation on P5. Some PCs in groups irradiated in the P5-P6 period were innervated by 5 CFs (Fig. 6), which corresponds to the maximum observed during early development^16^. If the hypothetical early phase of synapse elimination was GC-independent, the highly degranulating irradiation protocols on P5 should have had no influence on this early phase of synapse elimination and we should have seen only partial multiple innervation in the adult, due to interference with the later GC-dependent phase.

Many studies on mice lacking certain synaptic signaling proteins have been presented as supporting the existence of two phases of CF synapse elimination^13^. To take just one example, the deletion of PKCγ leads to CF multiple innervation in the adult knock-out mouse^24^. In these mice, CF synapse elimination appears normal in early development, and abnormalities are detected only from P15^24^, consistent with a first phase of CF elimination independent of PF synaptic activity. Alternatively, and more consistent with the data we present here, PC PKCγ is not highly expressed during the early stages of PF-PC synaptogenesis^40^ but its strong activity from P14 could contribute to later-stage synaptic refinement.

Although these signaling pathways linked to PF-PC synaptic activity are clearly required for the final period of synapse elimination^8^, less is known about mechanisms of the initial triggering phase. Climbing fiber activity-induced Ca^2+^ influx into the postsynaptic PCs^41^ as well as trophic factors^42–44^ are known to be critical for the early phase of synapse elimination. A plausible mechanism could involve a paracrine signal from GCs/PFs; or very early interactions with the first PF synapses could drive PC maturation, ultimately including CF synapse elimination.

This study emphasizes the importance of timing differences of neurogenesis between cerebellar regions, confirms the critical involvement of granule cell development in CF synapse elimination and indicates that all phases of CF synapse elimination, including its initiation, require the presence of granule cells.

### Ethical approval

All experiments were carried out in strict accordance with the national and international laws for laboratory animal welfare (NIH Publication 80–23, revised 1996 and European Parliament and Council Directive of September 22, 2010/63/EU) and were approved by the Comité Régional d’Ethique en Expérimentation Animale (Autorisation n° 67–163 Ministère de l’Enseignement Supérieur et de la Recherche, France).

## Materials and Methods

### Animals/Schedules of irradiation

Pregnant Wistar female rats were obtained from IFFA CREDO (Mérieux SA) and maintained at the local animal facility in Paris. Each litter was reduced to 8 pups on the day of birth (day 0). The posterior cerebellum of these newborn rats was irradiated with X-rays as previously described in detail^32^. Eleven schedules of irradiation were used (Table 1). The doses of irradiation are given in rads (r) instead of Grays (Gy) throughout the text to facilitate comparison with previous studies^28,32^. The results from these irradiation-schedule groups are shown in Table 2. Most of the X-irradiated rats survived and exhibited signs of transient ataxia which were much diminished by the adult stage (2 to 3 months) when electrophysiological experiments were carried out. No differences due to the age or the sex of the animals were found, in agreement with previous studies^28,32^.

### Intracellular electrophysiological recording of Purkinje cells *in vivo*

Adult rats were anaesthetized with ethyl carbamate (1 ml solution 10%,/100 g wt., i.p.), paralysed with gallamine triethiodide (80 mg/kg, i.p.) and artificially ventilated. Core temperature was maintained at 36–37 °C with a heating pad. The posterior cerebellar vermis was surgically exposed and covered with a gel of agar in 10% sucrose. Concentric bipolar electrodes (Rhodes Medical Instruments) were used to stimulate the climbing fiber pathway in the juxtafastigial region ipsilateral to the recording site (JF stimulation^18^). Intracellular recordings of spontaneous and evoked PC activity were performed with glass microelectrodes filled with 3 M potassium acetate (DC resistance 15–30 MΩ), along a sagittal band of the posterior vermis (500 µm from the midline on each side). All the tracks were in parallel sagittal planes. The relative position and depth of the Purkinje cells recorded were noted during the experimental sessions. The recordings were displayed on a storage oscilloscope and stored for further analysis. The number of CFs making synapses on each PC was estimated as previously described^18,32^.

In most PCs, the action potential disappeared early in the recording and only the underlying excitatory postsynaptic potentials (EPSPs), typical of the activation of the PCs through the CF pathway (CF-EPSPs), were recorded during several minutes at a stable resting potential between −30 to −50 mV. CF-EPSPs were evoked by JF stimulation of the CF pathway or occurred spontaneously. It was always possible to determine with great accuracy if a PC received one or more CFs according to the all-or-none or the stepwise graded character of the CF-EPSP (Fig. 1). We determined the index of innervation *m*, the number of steps in spontaneous and evoked CF EPSPs. This index of innervation *m* is the minimal estimate of the exact number of CFs establishing synapses on the PC^18,32^.

We analyzed 753 PCs from 10 different irradiation protocol groups (Table 2). In each experimental group, the majority of recorded cells were in lobules VII and VIII, although in some groups cells were also recorded in the anterior lobules (I-V) and the other posterior lobules (VI, IX and X). All parameters were calculated separately for each lobule. Both qualitative histological features and electrophysiological data were homogenous within each group^32^, indicating that the irradiation procedure was very reproducible from one animal to another, a crucial condition when only a few sequences of irradiation are used.

At the end of each experiment, an iontophoretic deposit of Fast green was made in two spots located 2 mm apart along the same track, and a small lesion was performed by applying current (150 µA, 20 s) through the stimulating electrode (Fig. 3a). The stereotaxic position of the entry of the recording electrode into the cerebellar cortex and the depth of the recorded PCs were used to reconstruct the different tracks of the microelectrodes and locate the PCs recorded in the different lobules on magnified (x 60) sagittal sections stained with Cresyl violet-thionin.

### Quantitative analysis of granule cells

Cresyl violet-thionin-stained cryostat sections from the vermis of cerebella analyzed in electrophysiology were used to measure the area of the GCL and the density of GCs within this region. Non-irradiated controls (n = 3) were compared to sections from irradiation Groups P5^200^, P5^600^, P5^800^, P6^600^, P7^600^ and P8^600^ (n = 3/group). GCL area was measured using Axiovision Rel 4.2 (Zeiss). Granule cell density was determined in areas of the internal granular layer (IGL) selected randomly (1 area/lobule) in each of all 13 lobules (I-V, VIa, VIb, VIc, VIIb, VIIIventral, VIIIdorsal, IXa, X) of the vermis of the cerebellar cortex. The stained nuclei were almost exclusively GCs (the large nuclei of Golgi interneurons stained weakly and were excluded from analysis). Ectopic GCs in the PC and molecular layer were also sampled. In these layers, light blue nuclei of basket and stellate interneurons and violet PCs were excluded from the analysis. The GC density was calculated as the area sampled in each lobule divided by the mean GC area, measured from 20 randomly selected stained cells.

### Calcium Binding D-28 K Protein (CaBP) immunohistochemistry for morphological analysis of Purkinje cells in P5^600^ X-irradiated rats

For morphological analysis of PCs, we performed immunohistochemistry for CaBP, a specific marker of cerebellar PCs^45^. Eight pups received a single dose of 600 r on day 5. The P5^600^ pups were sacrificed on P6, P8, P12 and P13 (n = 2 per age, 1 X-irradiated and 1 non-irradiated control). Under deep anaesthesia (ketamine 5% - xylazine 2%), the rats were perfused transcardially with 4% paraformaldehyde in 0.1 M phosphate buffer (PB), pH 7.2 at 4 °C. The brains were removed and immersed in 30% saccharose in PB for 2 days. Frozen sagittal sections (30 µm-thick) were cut from the cerebellar vermis using a cryomicrotome (Reichert, Austria). Anti-CaBP immunostaining with CaBP antiserum (1:5000, Bellinzona, Switzerland) was carried out on floating sections according to a classical peroxidase anti-peroxidase protocol^46^. The sections were examined with a Zeiss Axioskop microscope.

### Statistics

The number of CFs per PC is a discontinuous variable (taking values 1, 2, 3, 4 or 5), which precludes using parametric tests. Comparisons between groups (protocols, lobules, or irradiation doses) were performed with non-parametric tests: either the two-sample Wilcoxon test, or the Kruskal-Wallis test, followed when appropriate by the Steel-Dwass test for multiple comparisons. Proportions in contingency tables were compared with Fisher’s exact test for count data. Statistical analyses were performed with R (version 2.14.1, http://www.r-project.org) or with GraphPad Prism. Data are expressed as mean value ± standard error of the mean (S.E.M.).The significance level was 0.05.

Granule cell density was compared between the late- (VI, VII, VIII) and early- (I-V, IX-X) developing lobules in each experimental group and between groups using two-way (late- or early-developing lobules, groups) ANOVA followed by post hoc Tukey tests for multiple comparisons. The same statistical approach was used to compare the GCL area between the late- (VI, VII, VIII) and early- (I-V, IX-X) developing lobules with each experimental group, and between groups. The significance threshold was set at P = 0.01.

### Data availability

All data generated or analysed during this study are included in this published article.
